# Identification of potential key genes that respond to capsaicin treatment in ER-positive breast cancer: An integrated analysis

**DOI:** 10.1371/journal.pone.0350841

**Published:** 2026-06-03

**Authors:** Long Zheng, Wenjin Li, Bing Wang, Tao Wu, Hao Huang, Yongchao He, Wei Qu

**Affiliations:** Department of Nuclear Medicine, the Second Affiliated Hospital of Xi’an Jiaotong University, Xi’an, Shaanxi, China; Karpagam University: Karpagam Academy of Higher Education, INDIA

## Abstract

Capsaicin, a natural compound, has demonstrated antitumor efficacy in estrogen receptor-positive breast cancer (ER-positive BC). However, its downstream molecular targets and mechanisms remain poorly understood, particularly those linked to metabolic reprogramming and immune modulation. This study aimed to identify capsaicin-responsive genes and explore their roles in ER-positive BC progression and tumor microenvironment remodeling. Bioinformatic analysis was performed on the GSE64155 dataset from GEO to screen differentially expressed genes (DEGs) in capsaicin-treated ER-positive BC. Functional enrichment (GO/KEGG) and protein-protein interaction (PPI) network analyses were performed to prioritize key genes. Experimental validation included qPCR and Western blotting to assess gene and protein expression in capsaicin-treated cells. Clinical relevance was evaluated using TCGA expression data, survival analysis (overall survival [OS], relapse-free survival [RFS], and distant metastasis-free survival [DMFS]), and immunohistochemistry (IHC) in BC tissues. Immune cell infiltration was analyzed via the CIBERSORT algorithm. SHMT2 and GARS were identified as the most significant capsaicin-responsive genes. Both genes were overexpressed in BC tissues and may be associated with a poor prognosis in patients with ER-positive BC. Capsaicin significantly reduced SHMT2 and GARS expression at the mRNA and protein levels. Immune infiltration analysis indicated that high SHMT2 and GARS expression may be associated with altered immune cell infiltration patterns. This study identifies SHMT2 and GARS as novel downstream targets of capsaicin in ER-positive BC and links their known oncogenic functions to a potential new mechanism underlying the antitumor effects of capsaicin.

## Introduction

Based on data from the global tumor epidemiology in 2020, the numbers of new cases of breast cancer and related deaths (2,261,419 and 684,996, respectively) were the highest worldwide [1]. Currently, BC remains a global public health issue because of its continuously increasing morbidity, mortality, and economic burden. Previous research has shown that the estrogen receptor (ER) plays a crucial role in BC initiation and progression and is expressed in over70%−80% of BC tissues [2]. Compared with other BC subtypes, ER-positive BC is associated with better survival outcomes and longer disease-free survival [3]. The ER can bind to the estrogen response element (ERE) located in estrogen response genes and regulate their transcriptional activity when it is triggered by estrogens [4]. Drugs that can block the ER and prevent estrogenic stimulation, such as tamoxifen and letrozole, have become first-line therapies for BC. Therefore, we believe that estrogen response genes will become important therapeutic targets for BC and are valuable for further research.

Capsaicin is the active ingredient extracted from red peppers and is a phytochemical with broad-spectrum anticancer activity [5,6]. Chang et al. reported that mitochondrial dysfunction mediated the apoptosis induced by capsaicin in MCF-7 cells, which involved PARP-1 activation and apoptosis-inducing factor (AIF) release [7]. Another study revealed that capsaicin treatment resulted in G0/G1 cell cycle arrest, apoptosis, and cell migration through the inhibition of the protein expression of EGFR, HER-2, ERK, and cyclin D1 [8]. In addition, Lee et al. developed ^68^Ga-SCN-DOTA-Capsaicin as a radioactive imaging agent for targeting and promoting apoptosis and cell cycle arrest in breast cancer [9]. Compared with the single-drug administration of paclitaxel or capsaicin, a novel codelivery nanosystem developed by Lan et al. that combined paclitaxel and capsaicin using a prodrug micelle exhibited better synergistic antitumor activity [10]. Capsaicin can improve the antitumor activity of some chemotherapeutic drugs, such as docetaxel [11] and pirarubicin [6], when administered synergistically. In addition, Jankovic et al. reported that the dietary intake of capsaicin increased the PSA doubling time from 4 weeks to 7.3 months in a patient with castration-resistant prostate cancer [12].

Although the anti-breast cancer effects of capsaicin have been reported, its pharmacological effects and the underlying mechanisms of cancer prevention have not yet been fully elucidated. The rapid development of transcriptome data and bioinformatics analysis techniques has provided favorable conditions for further elucidating the landscape of the anticancer network of capsaicin. This study aimed to identify capsaicin-responsive genes in ER-positive breast cancer through an integrated transcriptomic analysis and preliminary experimental validation, thereby advancing our understanding of the therapeutic mechanisms of capsaicin.

## Materials and methods

### Reagents and antibodies

Capsaicin (M2028) was purchased from Sigma-Aldrich, diluted in DMSO, and stored as a 200 mmol/L stock solution. Anti-SHMT2 rabbit polyclonal antibody (#6347), anti-GARS rabbit polyclonal antibody (#7340), and anti-β-actin mouse monoclonal antibody were purchased from Affinity Biosciences. The TRIzol reagent, SuperScript III RT kit, and SYBR qPCR mix were purchased from Invitrogen.

### Cell culture

The human breast cancer cell line MCF-7 (ER-positive) was purchased from the China National Collection of Authenticated Cell Cultures (Shanghai, China) and cultured in RPMI 1640 (Gibco) supplemented with 10% fetal bovine serum (Evergreen, Hangzhou, China). The culture temperature was set at 37 °C, and the concentration of CO2 was maintained at 5%.

### Data source and description

The GSE64155 dataset, containing RNA-seq data of MCF-7 cells treated with capsaicin, was acquired from the GEO database (https://www.ncbi.nlm.nih.gov/geo/).

ER-positive breast cancer samples and normal breast tissues were obtained directly from the TCGA-BRCA project through the GDC Data Portal (https://portal.gdc.cancer.gov/). To ensure the relevance of ER-positive breast cancer, we first extracted ER status information from the TCGA clinical annotations, retaining only tumor samples explicitly labeled “ER-positive” (807 tumor samples). Normal breast tissue samples (79 samples) were identified on the basis of TCGA barcode classifications labeled “solid tissue normal.”

The Human Protein Atlas (HPA) serves as a comprehensive, open-access proteomic resource for systematically mapping the expression and spatial distribution of a vast majority of the human proteome (more than 26,000 human proteins). In this study, we utilized this platform to query the protein expression profiles of SHMT2 and GARS.

### Raw data normalization

The raw GSE64155 data were normalized by “normalizeBetweenArrays,” which is an R language command. It is a function used for data preprocessing in the limma package. Its core algorithm principle is based on quantile normalization. By calculating the quantile differences between samples, the data are normalized to the same distribution, thereby eliminating the batch effect. This function is often used in scenarios such as gene expression data that require cross-sample comparisons.

### Differentially expressed gene (DEG) analysis

DEGs were identified using the limma package in R (version 3.50.3) with a threshold of |log2 fold change| > 0.585 (equivalent to a fold change > 1.5) and a Benjamini-Hochberg adjusted P value < 0.05. This lenient fold-change threshold was selected to capture subtle but biologically relevant changes given the small sample size of the GSE64155 dataset (n = 3 replicates per group). All downstream analyses were based on the DEGs meeting both criteria. The heatmap plot was generated using R software.

### Gene Ontology (GO) and Kyoto Encyclopedia of Genes and Genomes (KEGG) pathway enrichment analyses

To identify significantly enriched biological functions and pathways, we conducted functional enrichment analysis for GO terms and KEGG pathways using the Annotation, Visualization, and Integrated Discovery (DAVID) bioinformatics resource. The enrichment results, as computed and provided by the DAVID platform, were subsequently used to generate graphical representations. Specifically, bar plots and dot plots were created using R software, directly utilizing functional categories obtained from the analysis.

### Protein-protein interaction (PPI) network analysis

The PPIs were investigated using the STRING database. Initial interaction data for the proteins of interest were retrieved from STRING. This primary interaction dataset was then subjected to network analysis and visualization using the open-source software platform Cytoscape, which facilitated the generation of a comprehensive PPI network diagram. The MCODE plugin was run with default settings: node score cutoff = 0.2, k-core = 2, and max depth = 100. Clusters were ranked by connectivity, and the top-scoring module was selected.

### Expression analysis and survival analysis

RNA expression analysis of candidate genes was conducted using the TCGA database. RNA expression data of ER-positive BC were screened out by Perl software, and differential expression analysis was conducted using R packages.

Survival analysis was conducted using ER-positive breast cancer cohorts on the KM plotter website. Median expression was used to stratify high/low groups, with log-rank tests for survival curves (assumptions of proportional hazards were not formally tested owing to tool limitations). The limitation is that no adjustments for clinical covariates (such as age or tumor stage) were performed, as KM plotter provides precurated data.

### CIBERSORT analysis

To deconvolute the immune cell composition within the tumor microenvironment, we employed CIBERSORT, an analytical tool designed to infer the relative abundances of specific cell subsets from bulk tissue gene expression profiles. ER-positive breast cancer samples were stratified into high- and low-expression cohorts on the basis of the median expression levels of SHMT2 and GARS. The relative infiltration levels of 22 distinct immune cell subtypes were subsequently estimated for each cohort. The differential infiltration patterns between groups were visualized using violin plots, which were generated with R software.

### Total RNA isolation, reverse transcription, and quantitative real-time polymerase chain reaction (qPCR)

Total RNA from the MCF-7 cells was extracted and quantified by measuring its absorbance at 260 nm using a microplate autoreader (Bio-Tek Instruments, Inc.). RNA (500 ng) was reverse transcribed. Quantitative real-time PCR (qPCR) was then performed using Applied Biosystems. The experimental steps were carried out according to our previously published methods [13]. For the qPCR process, the conditions included predenaturation at 95 °C for 30 sec for one cycle, followed by PCR at 95 °C for 5 sec and 60 °C for 30 sec for 40 cycles, and a third stage of dissociation at 95 °C for 15 sec followed by 60 °C for 30 sec and 95 °C for 15 sec. β-actin was used as the internal control, and the primers used for qPCR are shown in S1 Table.

### Western blot

Capsaicin-treated MCF-7 cells were harvested and washed with cold PBS three times. All Western blot experiments were performed with three independent biological replicates. Total cellular protein lysates were treated with radioimmunoprecipitation assay buffer (50 mM Tris (pH 8.0), 150 mM NaCl, 0.1% SDS, 1% Nonidet-P40, and 0.5% sodium deoxycholate) supplemented with proteinase inhibitors (1% cocktail and 1 mM phenylmethanesulfonyl fluoride, both from Sigma-Aldrich). Afterward, the lysates of the MCF-7 cells were centrifuged at 20,000 × g for 15 min at 4 °C, after which the supernatants were collected. The protein concentration was quantified using an Enhanced BCA Protein Assay Kit (Beyotime Institute of Biotechnology, Haimen, China). Subsequent Western blot analysis was performed according to the experimental steps applied in our previous work [6]. For Western blot analysis, the protein lysates were separated by SDS-PAGE and transferred to nitrocellulose membranes. After blocking in 5% nonfat dry milk, the membranes were probed with specific primary antibodies overnight at 4 °C. After being washed three times with TBS, the membranes were incubated with secondary antibodies at room temperature in the dark for 1 h, followed by washing as above in the dark, drying with neutral absorbent paper, and scanning using an Odyssey detection system (LI-COR). β-actin was used as the loading control.

### Statistical analysis

One-way ANOVA was used to assess the statistical significance of the CCK-8 and qPCR data. Statistical significance was set as a *P* value < 0.05.

## Results

### Raw data normalization

**Fig 1:**
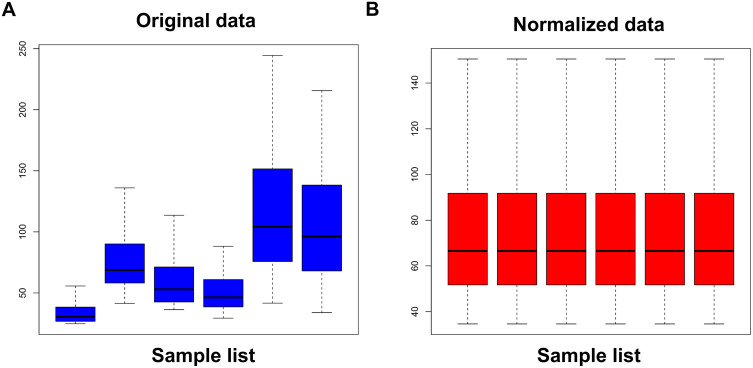
Normalization of gene expression data in samples of GSE64155. **(A)** Before normalization **(B)** After normalization. The blue bar represents the data before normalization, and the red bar represents the data after normalization.

The expression data in the GSE64155 dataset include triplicate quantified RNA expression values of MCF-7 cells grouped into the control and capsaicin treatment groups. The raw expression data were successfully normalized by the “normalizeBetweenArrays” command in the R language (Fig 1).

### Identification of DEGs between the capsaicin treatment group and control group in GSE64155

**Fig 2:**
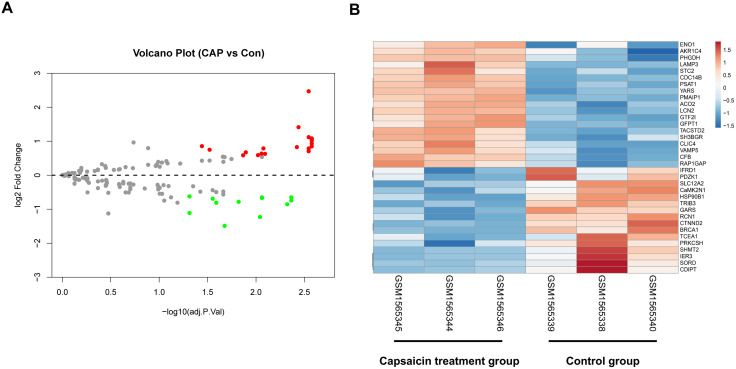
DEGs were identified based on the data of GSE64155. **(A)**. Volcano plot of DEGs between the capsaicin treatment group and the control group. Red points represent up-regulated genes, and green points represent down-regulated genes. Genes with no significant difference are shown in gray. **(B)**. Heatmap of the identified DEGs. Up-regulated genes are shown in red, while down-regulated genes are shown in green.

Genes with expression fold changes > 1.5 (|log2 fold change| > 0.585) and an adjusted P value < 0.05 were considered to indicate a DEG. A total of 32 DEGs, 19 of which were upregulated and 13 of which were downregulated, were identified by the limma R package. The clusters of all DEGs are shown in a volcano plot (Fig 2A). The pheatmap R package was used to construct the heatmap plot, visually representing all DEGs in the control and capsaicin-treated samples (Fig 2B). Detailed information on all DEGs is shown in S2 Table.

### GO and KEGG enrichment analyses of genes related to capsaicin treatment

**Fig 3:**
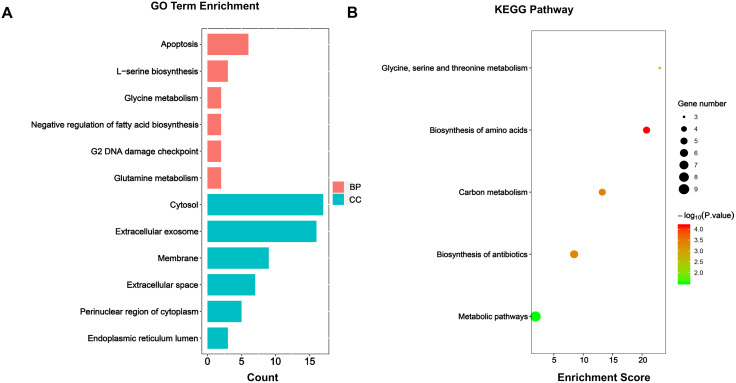
GO and KEGG enrichment analyses of DEGs. **(A)** GO analysis divided DEGs into two groups as follows: biological processes (red bars), cell components (blue bars). **(B)** Bar plot of KEGG pathway enrichment analysis. BP, biological processes; CC, cell components.

The GO terms of the DEGs were clustered into three groups: biological process, molecular function, and cellular component. As shown in Fig 3A, the DEGs in our study were enriched in biological processes (BPs) and cellular components (CCs). In terms of biological processes, these genes were abundantly enriched in apoptosis, L-serine biosynthesis, glycine metabolism, negative regulation of fatty acid biosynthesis, the G2 DNA damage checkpoint, and glutamine metabolism. We also subjected the DEGs to KEGG pathway enrichment analysis. As shown in Fig 3B, the involved pathways were enriched mainly in metabolic pathways, the biosynthesis of antibiotics, carbon metabolism, the biosynthesis of amino acids, and glycine, serine, and threonine metabolism.

### SHMT2 and GARS were identified as potential key genes that respond to capsaicin treatment

**Fig 4:**
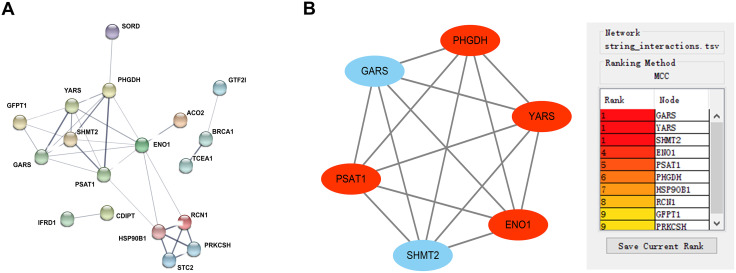
PPI network and hub genes in the capsaicin treatment groups. **(A)** PPI network. **(B)** Hub genes and interaction in the capsaicin treatment groups. ‌‌Circles represent genes, and lines represent interactions among DEGs.

All 32 DEGs were input into the STRING database. The PPI network (Fig 4A) and primary protein interaction data were generated automatically. Two downregulated genes and four upregulated genes were identified as hub genes by MCODE analysis (Fig 4B). The downregulated hub genes were SHMT2 and GARS, and the upregulated genes were ENO1, PHGDH, PSAT1, and YARS.

According to the MCC ranking, three genes (GARS, YARS, and SHMT2) ranked first. The results of the literature search indicated that YARS is a newly identified gene with uncertain functions in breast cancer since no systematic experimental research has been performed. Thus, we selected SHMT2 and GARS as potential key capsaicin-responsive genes for further investigation. This identification represents a novel association, linking these oncogenic markers to capsaicin treatment in ER-positive BC.

### Validating the effect of capsaicin on expression profiles of SHMT2 and GARS

**Fig 5:**
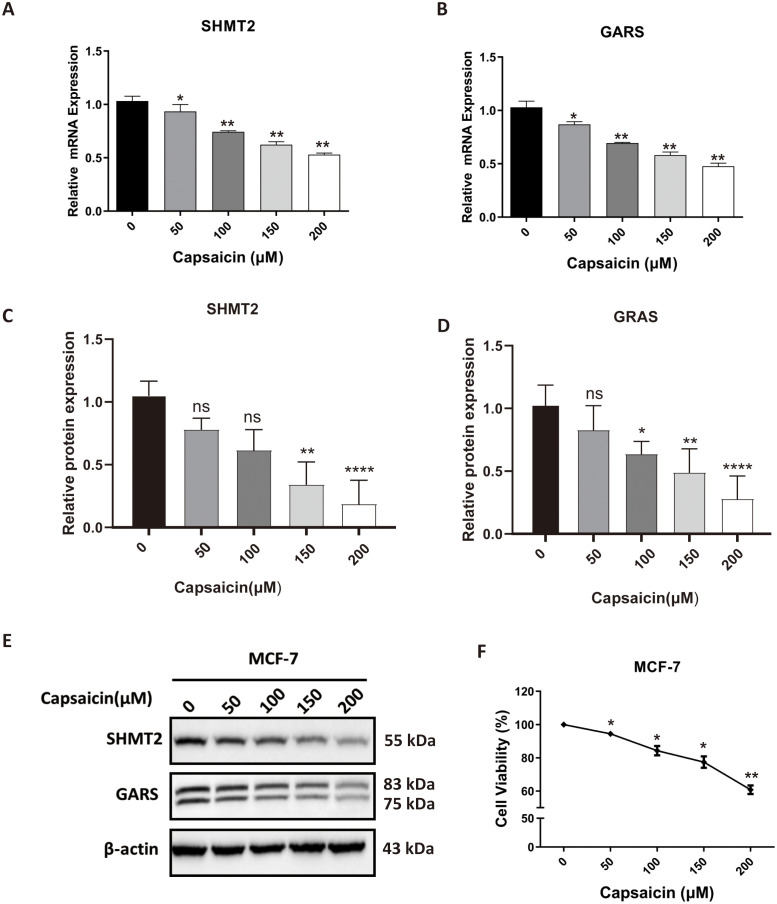
Validating the effect of capsaicin on expression profiles of SHMT2 and GARS. **(A and B)** Capsaicin inhibited the mRNA expression of SHMT2 and GARS. **(C and D)** Densitometric quantification of SHMT2 and GARS protein levels from Western blots (normalized to β‑actin). **(E)** The protein expression of SHMT2 and GARS was decreased by capsaicin treatment. **(F)** The CCK-8 assay shows that capsaicin induced growth inhibition of MCF-7 cells. All experiments were performed with three independent biological replicates (n = 3). Data are mean ± SD. One‑way ANOVA with Dunnett’s post‑hoc test was used for multiple comparisons vs. control. *P < 0.05, **P < 0.01, ***P < 0.001, ****P < 0.0001.

To further explore the effect of capsaicin on SHMT2 and GARS expression, we performed CCK-8, qPCR, and Western blot analyses. The qPCR results revealed that capsaicin treatment significantly reduced the mRNA levels of both SHMT2 and GARS in a concentration-dependent manner (Fig 5A and B). Western blot analysis was performed with three independent biological replicates. Densitometric quantification revealed that at concentrations of 150 µM and 200 µM, capsaicin significantly decreased SHMT2 protein expression (Fig 5C) and similarly reduced GARS protein expression at all tested concentrations (Fig 5D). Representative Western blot images are shown in Fig 5E. Consistent with these molecular changes, the results of the CCK-8 assay demonstrated that capsaicin inhibited MCF-7 cell viability in a dose-dependent manner (Fig 5F).

### Validating the expression profiles of SHMT2 and GARS in the TCGA and HPA databases

**Fig 6:**
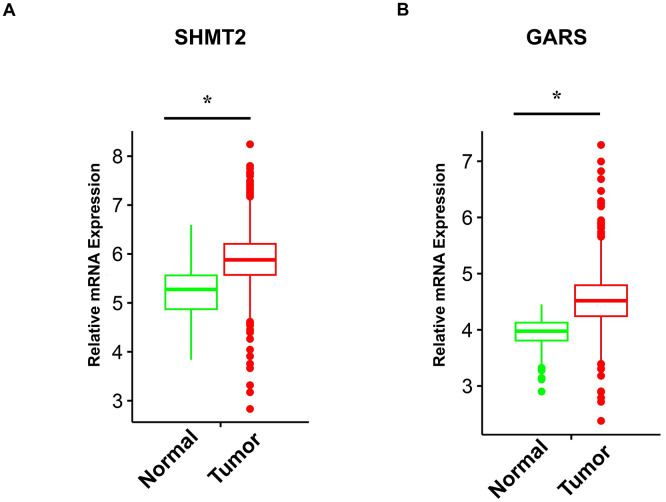
The mRNA expression of SHMT2 and GARS was elevated in ER-positive BC samples. The RNA-seq data were screened from the TCGA database. Statistical analysis was performed using unpaired two-tailed Student’s t-test. *P < 0.05.

**Fig 7:**
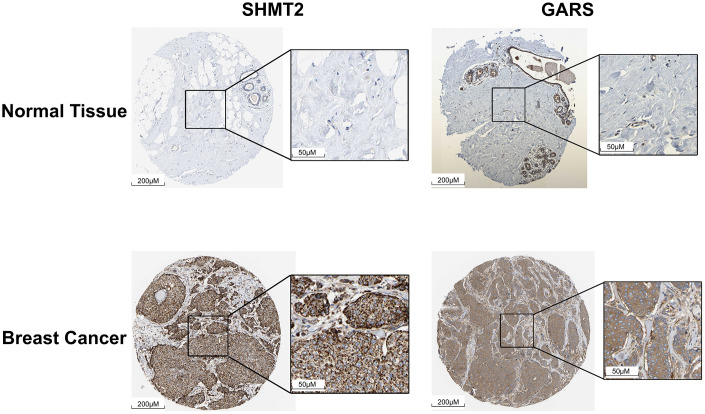
SHMT2 and GARS proteins were highly expressed in BC tissues. The IHC data were acquired from the HPA database.

On the basis of the data from the TCGA database, we screened 79 normal samples and 807 ER-positive BC samples. The results indicated that the mRNA expression of SHMT2 and GARS in ER-positive BC tissues was significantly greater than that in normal tissues (Fig 6). Consistent with these findings, compared with normal tissues, the protein expression of both genes in the HPA database was also markedly elevated in BC tissues (Fig 7).

### High expression of SHMT2 and GARS may be associated with poor clinical outcomes in ER-positive patients

**Fig 8:**
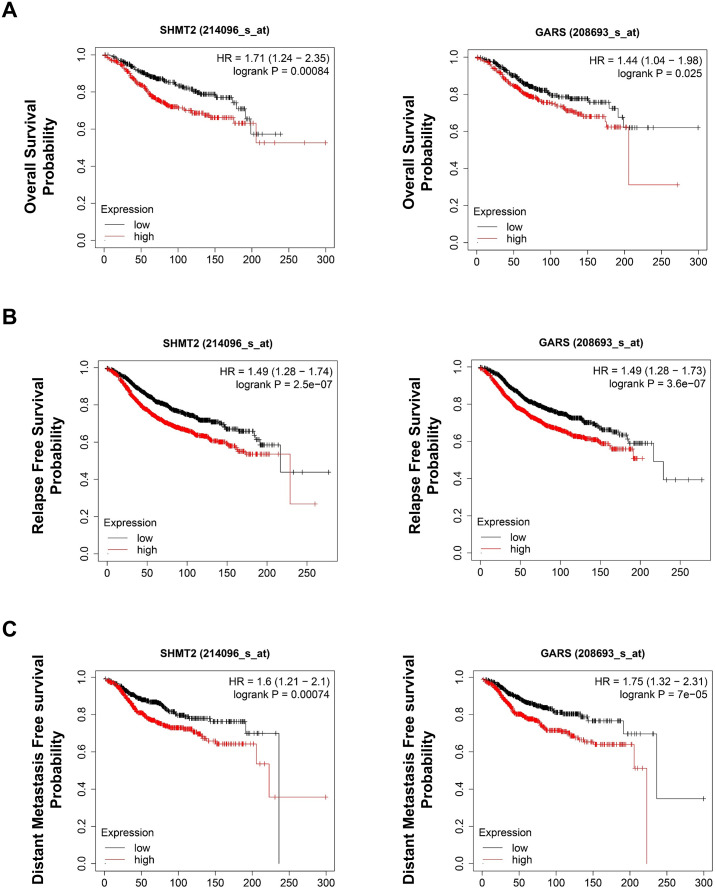
Kaplan-Meier survival analysis of SHMT2 and GARS in ER-positive BC. **(A)** Overall survival, OS; **(B)** Relapse-free survival, RFS; **(C)** Distant metastasis-free survival, DMFS.

To research the prognostic value of SHMT2 and GARS, we conducted survival analysis in the KM plotter database, which is a website designed to evaluate the survival of more than 54,000 genes in 21 cancer types. As shown in Fig 8, high expression of the two genes may be associated with a poor prognosis, including shorter OS, RFS, and DMFS, in patients with ER-positive BC. It should be noted that no adjustments for important clinical covariates were performed, as KM plotter provides pre-curated, univariate analyses. This lack of adjustment may introduce confounding biases, potentially overestimating the prognostic associations of SHMT2 and GARS. These limitations require validation through multivariable models in future studies.

### SHMT2 and GARS are associated with immune cell infiltration status in ER-positive BC

The immune system can play a dual role in breast cancer by either promoting or inhibiting tumor growth and progression, depending on the type of immune cells and the disease stage. Previous studies have proven that immune cell infiltration within the breast cancer microenvironment can significantly affect disease progression and patient prognosis.

In the present study, we applied the CIBERSORT algorithm to further explore the correlation between the subtypes of immune cells and the expression profiles of the two identified genes (SHMT2 and GARS). The immune infiltration scores of a total of 22 subtypes of immune cells were calculated in the high- and low-gene expression groups.

**Fig 9:**
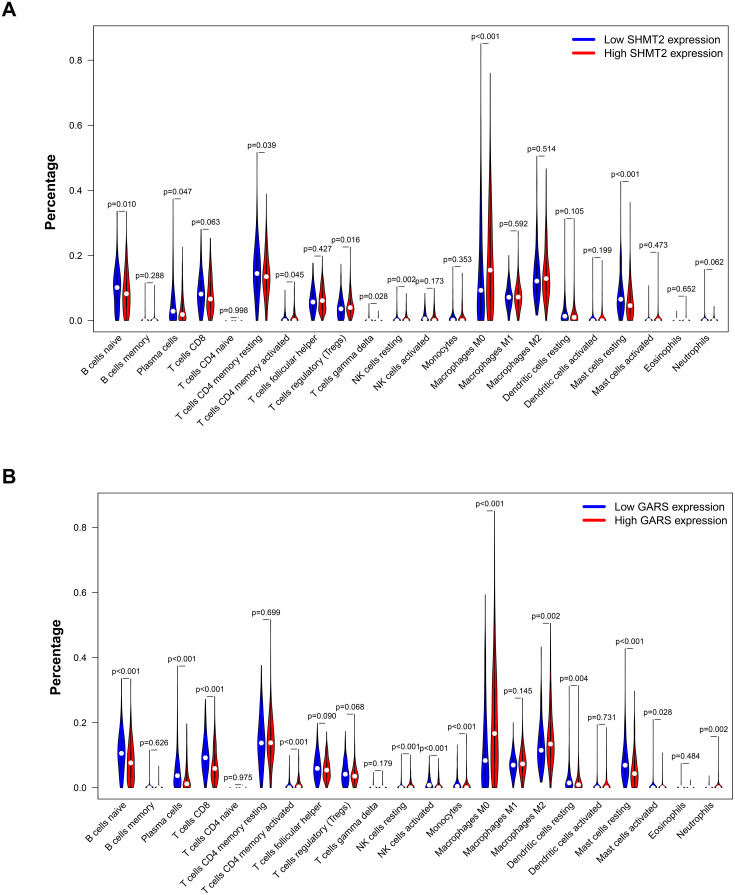
Immune cell infiltration analysis by CIBERSORT algorithm. **(A)** The relationship between different groups of SHMT2 expression and immune cell infiltration. **(B)** The infiltration levels of immune cell subtypes in the high and low expression of the GARS groups.

High expression of SHMT2 may be significantly associated with low infiltration levels of naive B cells, plasma cells, CD8 + T cells, γδ T cells, and activated NK cells and high infiltration levels of regulatory T cells, resting NK cells, M0 macrophages, and eosinophils (Fig 9A). Similarly, high GARS expression may be negatively associated with the infiltration of naive B cells, plasma cells, CD8 + T cells, follicular helper T cells, activated NK cells, monocytes, and resting dendritic cells and positively associated with the infiltration of resting NK cells, M0 macrophages, M2 macrophages, and neutrophils (Fig 9B). These patterns indicate potential associations between SHMT2/GARS expression and immune cell infiltration levels, warranting‌‌ further investigation.

## Discussion

Although capsaicin has been proven to be an effective anticancer drug, the mechanisms underlying the effects of capsaicin treatment on ER-positive BC remain unclear. Our study identifiesSHMT2 and GARS as potential capsaicin-responsive genes in ER-positive BC, generating hypotheses about their involvement in capsaicin’s antitumor effects. While the oncogenic roles of SHMT2 and GARS in BC are already well established based on prior research, our findings highlight their downregulation by capsaicin as a novel observation that warrants further mechanistic investigation.

As the key enzyme that links the serine/glycine synthesis pathway and one-carbon metabolism, serine hydroxymethyltransferase-2 (SHMT2) functions in the conversion of serine to glycine and provides a one-carbon unit to the folate and methionine cycles [14]. High expression or abnormal activation of SHMT2 has been proven to be a powerful oncogenic driver in diverse cancer types [15], such as lymphoma [16], gastric cancer [17], bladder cancer [18], and lung cancer [19]. With respect to BC, previous studies have revealed that the SHMT2 expression level is positively correlated with tumor clinical characteristics (TNM stage and clinical grade) [20,21]. Xie et al. reported that SHMT2 could trigger VEGF and MAPK signaling to promote breast cancer growth [22]. Qi et al. reported that the upregulation of SHMT2 expression by circ_0072995 promoted malignant cell phenotypes and anaerobic glycolysis in breast cancer [23]. Another study by Bernhardt et al. revealed that a high SHMT2 level predicts poor overall survival in patients with BC [24]. Because SHMT2 is an important estrogen response gene, its activation by ERα has been shown to mediate resistance to lapatinib in ER-positive BC [25]. Consistent with HPA database analyses (Fig 7), SHMT2 expression was elevated in BC tissues compared to normal tissues, and higher levels were associated with poorer prognosis (Fig 8). Our findings aligned with those of previous reports on the oncogenic role of SHMT2 in BC. These observations suggest that the capsaicin-induced downregulation of SHMT2 expression may disrupt serine/glycine synthesis and one-carbon metabolism, potentially contributing to antitumor effects. However, this remains a hypothesis that requires validation in future experimental studies.

Although SHMT2 has been predicted to be a potential oncogenic driver with prognostic value, only a few studies have reported its potential mechanisms of action. STAT3 is an upstream transcription factor that can bind to the promoter and induce the overexpression of SHMT2 under hypoxic or oxidative stress conditions. IL-6 triggers JAK/STAT3 signaling, upregulates SHMT2, and enhances energy metabolism in prostate cancer cells [26]. Additionally, Wang et al. reported that the overexpression of SHMT2 increased glycine synthesis and Akt/mTOR pathway activity, which promoted liver regeneration after partial hepatectomy. Because STAT3 and Akt/mTOR are proven targets of capsaicin, their inactivation is associated with the anticancer effects of capsaicin [27]. Our present study revealed that the administration of capsaicin at different concentrations significantly decreased SHMT2 expression in a dose-dependent manner. Thus, we hypothesize that the STAT3 and Akt/mTOR pathways may be involved in the downregulation of SHMT2 and mediate the effects of capsaicin on estrogen receptor signaling in ER-positive BC. However, these connections are speculative and require direct experimental validation in future research, such as via promoter assays, pathway inhibitors, or knockdown or overexpression experiments.

Glycyl-tRNA synthetase (GARS) is an indispensable enzyme for protein synthesis in mammalian cells. Previous studies have demonstrated that abnormal activity of GARS caused by gene mutation is strongly associated with the initiation of hereditary peripheral neuropathies, such as Charcot-Marie-Tooth disease [28], distal hereditary motor neuropathy V [29], and infantile spinal muscular atrophy [30]. Additionally, several studies have reported other functions of GARS outside the field of neuropathy. Park et al. reported that injury signal-triggered GARS production could increase the proliferation, differentiation, and migration of mesenchymal stem cells via the cadherin-6-mediated activation of the PI3K/Akt and FAK/ERK1/2 pathways in tissue repair [31]. Another study by Mo et al. revealed that GARS could promote cullin neddylation by binding to the ubiquitin-like protein NEDD8, thereby enhancing p27 degradation and promoting cell cycle progression in HeLa cells [32]. They also reported that high GARS expression was correlated with shorter DFS in patients with BC [32]. Yano et al. identified GARS as a potential target for overcoming gefitinib resistance in prostate cancer cells [33].

High GARS expression was associated with poor OS and DFS, which partly aligned with the findings of the study by Mo et al. [32]. In addition, our study supported the potential prognostic value of this gene and generated the hypothesis that the capsaicin-mediated downregulation of GARS expression may impair protein synthesis processes, a possibility that merits further exploration. We also speculated that the downregulation of GARS by capsaicin could disrupt PI3K/Akt signaling. However, the detailed mechanisms through which GARS acts in capsaicin-treated BC cells remain unclear and require further research.

CIBERSORT analyses (Fig 9) indicated associations between high SHMT2 or GARS expression and altered immune infiltration in ER-positive BC, including reduced antitumor cells (e.g., CD8 + T cells, activated NK cells) and increased pro-tumor cells (e.g., regulatory T cells, M2 macrophages). These findings suggested that SHMT2 and GARS were associated with an altered tumor microenvironment in ER-positive BC. Similarly, Kong et al. reported that high SHMT2 expression was associated with a lower immune score and that SHMT2 expression was positively correlated with the infiltration of Tregs. They demonstrated that SHMT2 could induce immunosuppression in papillary renal cell carcinoma [34]. A recent study of hepatocellular carcinoma conducted by Wang et al. revealed that elevated GARS expression enhanced CD206 + M2 macrophage infiltration in vivo [35]. Given that SHMT2 and GARS were identified as capsaicin-responsive genes, we hypothesized that capsaicin could modulate immune infiltration by downregulating these genes. However, some limitations should be mentioned. The present study relied on computational analysis via CIBERSORT for immune microenvironment analysis but lacked direct experimental validation (e.g., immunohistochemistry or flow cytometry). Consequently, the immune-related findings are presented as correlations rather than causal mechanisms. Future in vitro or in vivo immune assays should be performed to validate these associations and explore the immunomodulatory effects of capsaicin.

## Conclusion

In the current study, we identified SHMT2 and GARS as the two potential capsaicin-responsive genes in ER-positive BC through an integrated analysis and experimentally confirmed that capsaicin could downregulate SHMT2 and GARS expression. While their oncogenic roles in BC are already well established, this identification generates hypotheses regarding their involvement in capsaicin’s antitumor effects. Overall, we hypothesized that SHMT2 and GARS may serve as potential downstream targets involved in the antitumor effects of capsaicin in ER-positive BC. Further experimental studies, such as biological and in vivo experiments, are needed to validate these hypotheses and explore the detailed mechanisms involved.

## Supporting information

S1 Table The primers used in qPCR. (DOCX)

S2 Table Differentially expressed genes (DEGs) in GSE64155. Genes with expression fold change >1.5 (|logFC| > 0.585) and P < 0.05 were considered as DEGs.
(DOCX)

S1 File The original images for the western blot assay. Three independent experiments (Rep 1, Rep 2, Rep 3) were performed using separately cultured cells.
(ZIP)

S2 File The original data of GSE 64155 and immune infiltration analysis. (ZIP)
