# Supplementary material for: Identification of potential key genes that respond to capsaicin treatment in ER-positive breast cancer: An integrated analysis
Source: PLoS One. 2026 Jun 3;21(6):e0350841. doi: 10.1371/journal.pone.0350841 (PMC13232819; doi:10.1371/journal.pone.0350841)
Supplement: S1 Table — (DOCX) [file pone.0350841.s001.docx]

Table 1. The Primers used in qPCR

| Target Name | Primer | |
| --- | --- | --- |
| β-actin | Forward | 5’-TCCTCCTGAGCGCAAGTACTCC-3’ |
|  | Reverse | 5’-CATACTCCTGCTTGCTGATCCAC-3’ |
| SHMT2 | Forward | 5’-ATTCCCTCGCCTTTCAAGCAC-3’ |
|  | Reverse | 5’-CAGCCTTCACCCCTTTCCGGTA-3’ |
| GARS | Forward | 5’-GATGTGCTGATCGTTCCTGT-3’ |
|  | Reverse | 5’-TATATGCCTTACCAATTGCTCCC-3’ |
