# Supplementary material for: Identification of potential key genes that respond to capsaicin treatment in ER-positive breast cancer: An integrated analysis
Source: PLoS One. 2026 Jun 3;21(6):e0350841. doi: 10.1371/journal.pone.0350841 (PMC13232819; doi:10.1371/journal.pone.0350841)
Supplement: S2 Table — Genes with expression fold change >1.5 (|logFC| > 0.585) and P < 0.05 were considered as DEGs. (DOCX) [file pone.0350841.s002.docx]

**Table 2 Differentially expressed genes (DEGs) in GSE64155.** Genes with expression fold change >1.5 (|logFC|>0.585) and adj.P.Val <0.05 were considered as DEGs.

| Gene | LogFC | adj.P.Val |
| --- | --- | --- |
| LCN2  CDC14B  YARS  TACSTD2  CFB  PSAT1  CLIC4  PMAIP1  ACO2  VAMP5  GTF2I  TRIB3  GARS  RCN1  GFPT1  RAP1GAP  CTNND2  BRCA1  HSP90B1  PHGDH  SLC12A2  SH3BGR  LAMP3  STC2  CaMK2N1  PRKCSH  SHMT2  IER3  AKR1C4  ENO1  CDIPT  IFRD1 | 1.062978347  0.931117161  0.849597525  1.014651923  1.082688334  0.787925505  2.468844537  0.706008554  1.119972843  1.41208625  0.826386471  -0.65157765  -0.742379394  -0.855306949  0.629500811  0.787869629  -0.667349597  -0.652054  -0.668752281  0.635618824  -1.228189349  0.590514611  0.673386872  0.58560594  -0.780879828  -1.489027326  -0.809002572  -0.689919677  0.749649095  0.855150656  -0.622877001  -1.111297871 | 0.002655419  0.002655419  0.002655419  0.002655419  0.002655419  0.002858198  0.002858198  0.002858198  0.002858198  0.003635911  0.003784395  0.004322476  0.004322476  0.004761123  0.008069554  0.008382679  0.008689157  0.008689157  0.008689157  0.008766829  0.009094207  0.009539058  0.012752324  0.013588113  0.015104863  0.021105773  0.025749045  0.028036566  0.030150452  0.036215117  0.048656442  0.048656442 |
