# Supplementary figures and images for: Identification of potential key genes that respond to capsaicin treatment in ER-positive breast cancer: An integrated analysis
Source: PLoS One. 2026 Jun 3;21(6):e0350841. doi: 10.1371/journal.pone.0350841 (PMC13232819; doi:10.1371/journal.pone.0350841)

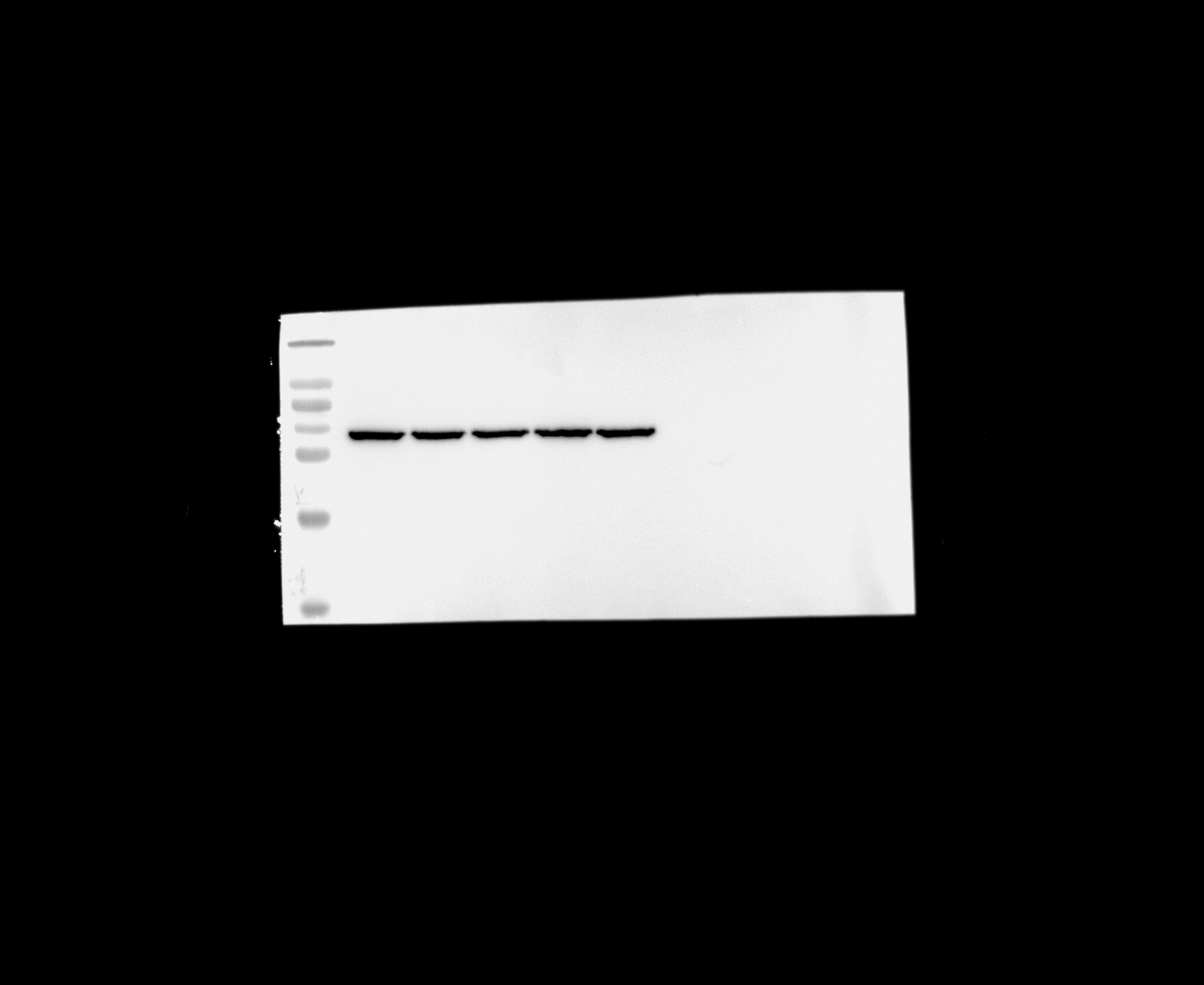

Supplement: S1 File — Three independent experiments (Rep 1, Rep 2, Rep 3) were performed using separately cultured cells. (ZIP) [file pone.0350841.s003.zip › data process/The first biological repetition/Actin.tif]

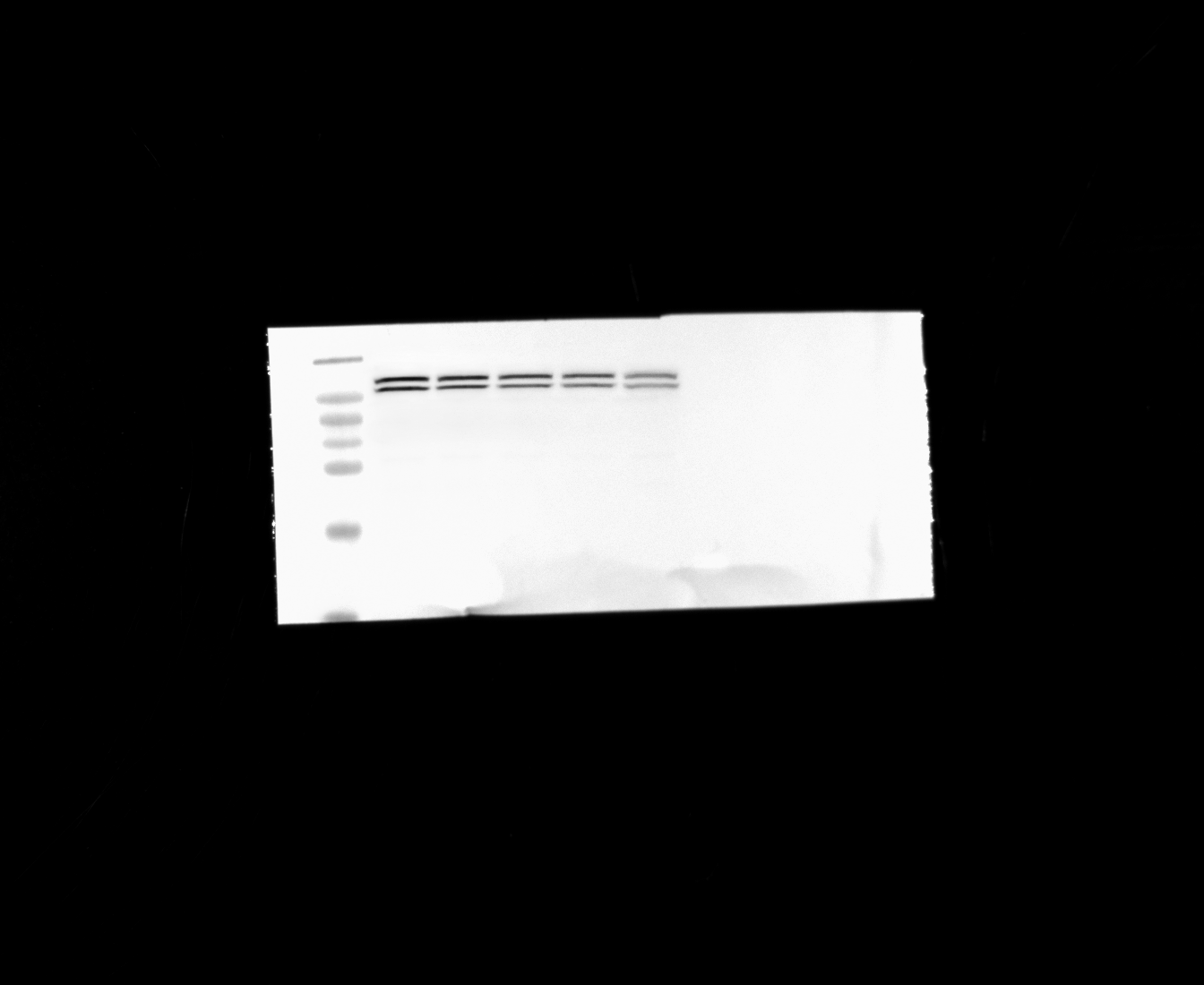

Supplement: S1 File — Three independent experiments (Rep 1, Rep 2, Rep 3) were performed using separately cultured cells. (ZIP) [file pone.0350841.s003.zip › data process/The first biological repetition/GARS.tif]

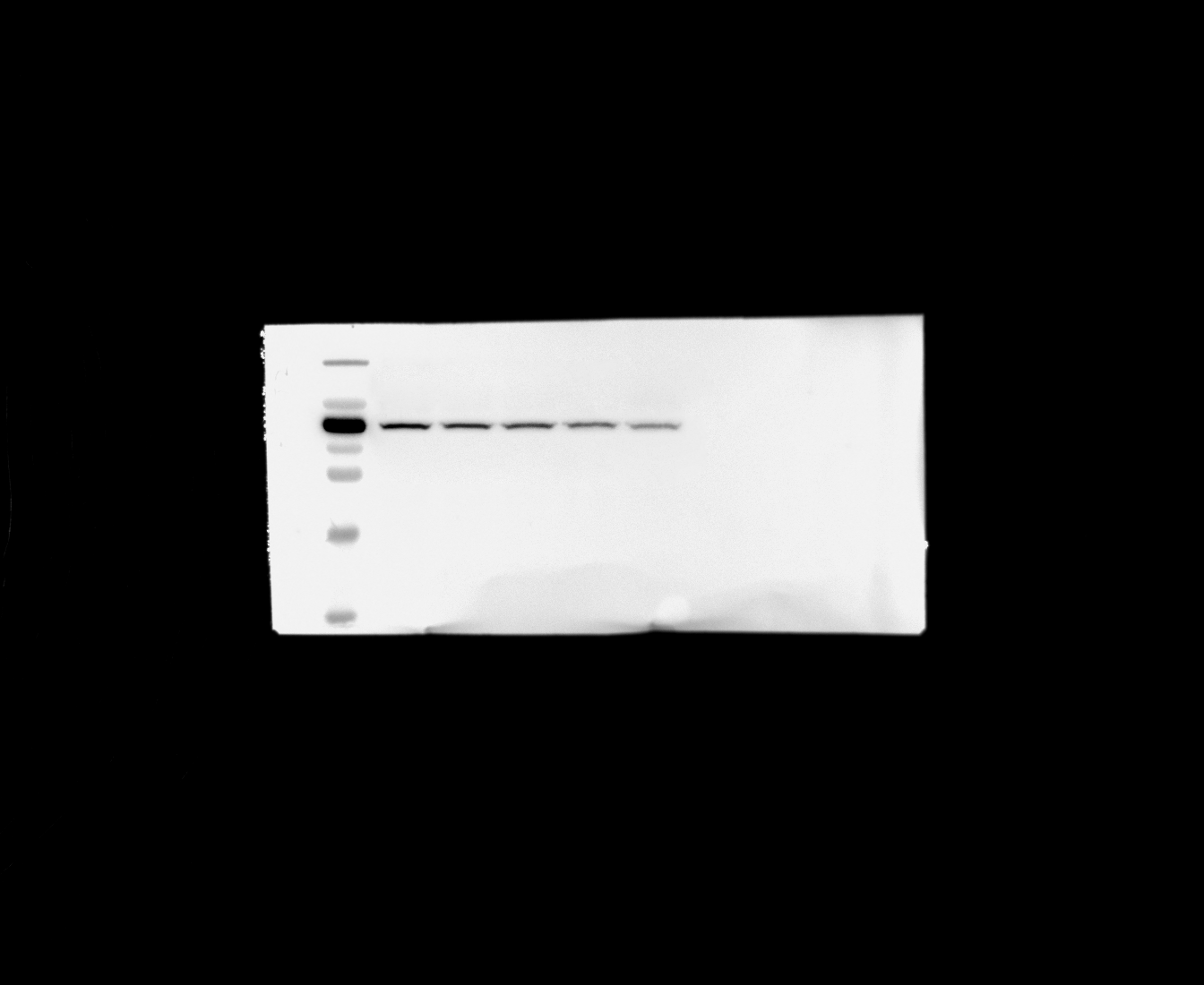

Supplement: S1 File — Three independent experiments (Rep 1, Rep 2, Rep 3) were performed using separately cultured cells. (ZIP) [file pone.0350841.s003.zip › data process/The first biological repetition/SHMT2.tif]

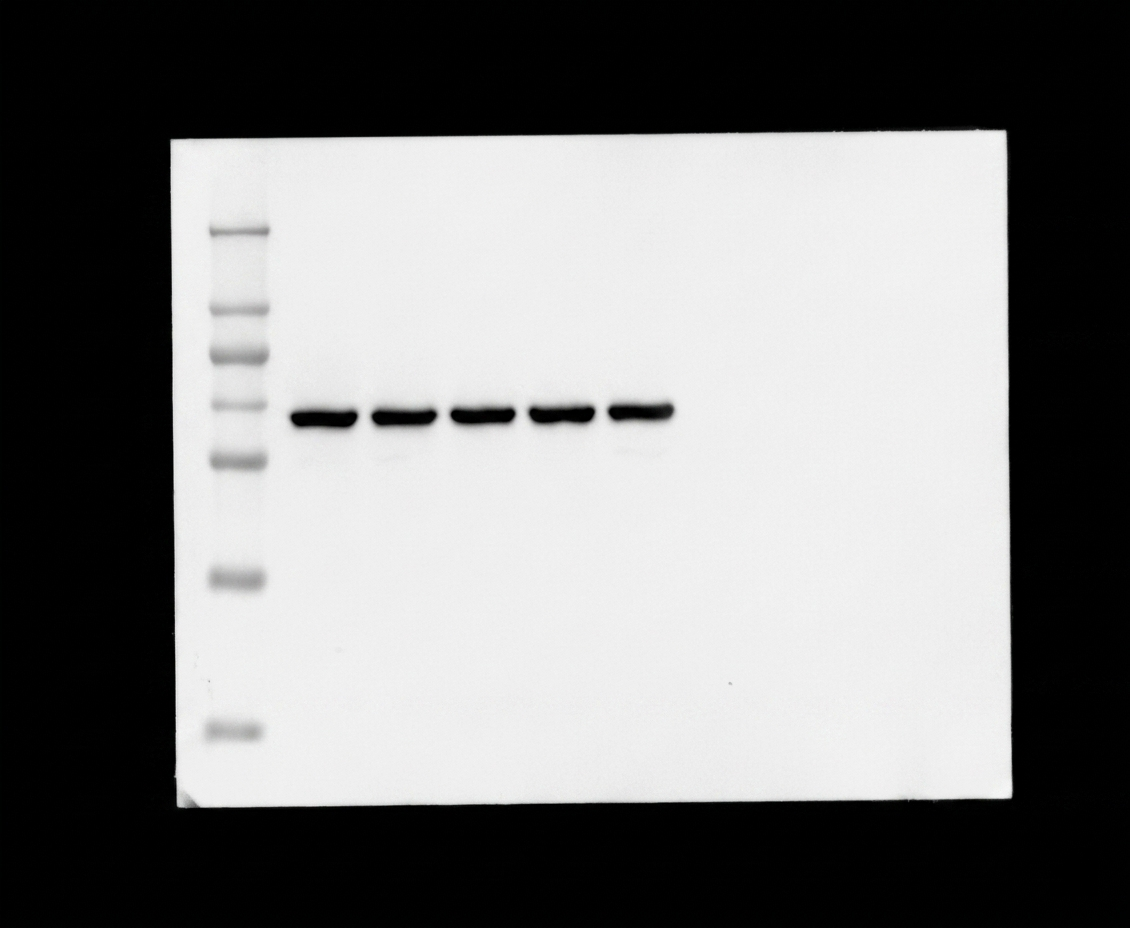

Supplement: S1 File — Three independent experiments (Rep 1, Rep 2, Rep 3) were performed using separately cultured cells. (ZIP) [file pone.0350841.s003.zip › data process/The second biological repetition/actin.tif]

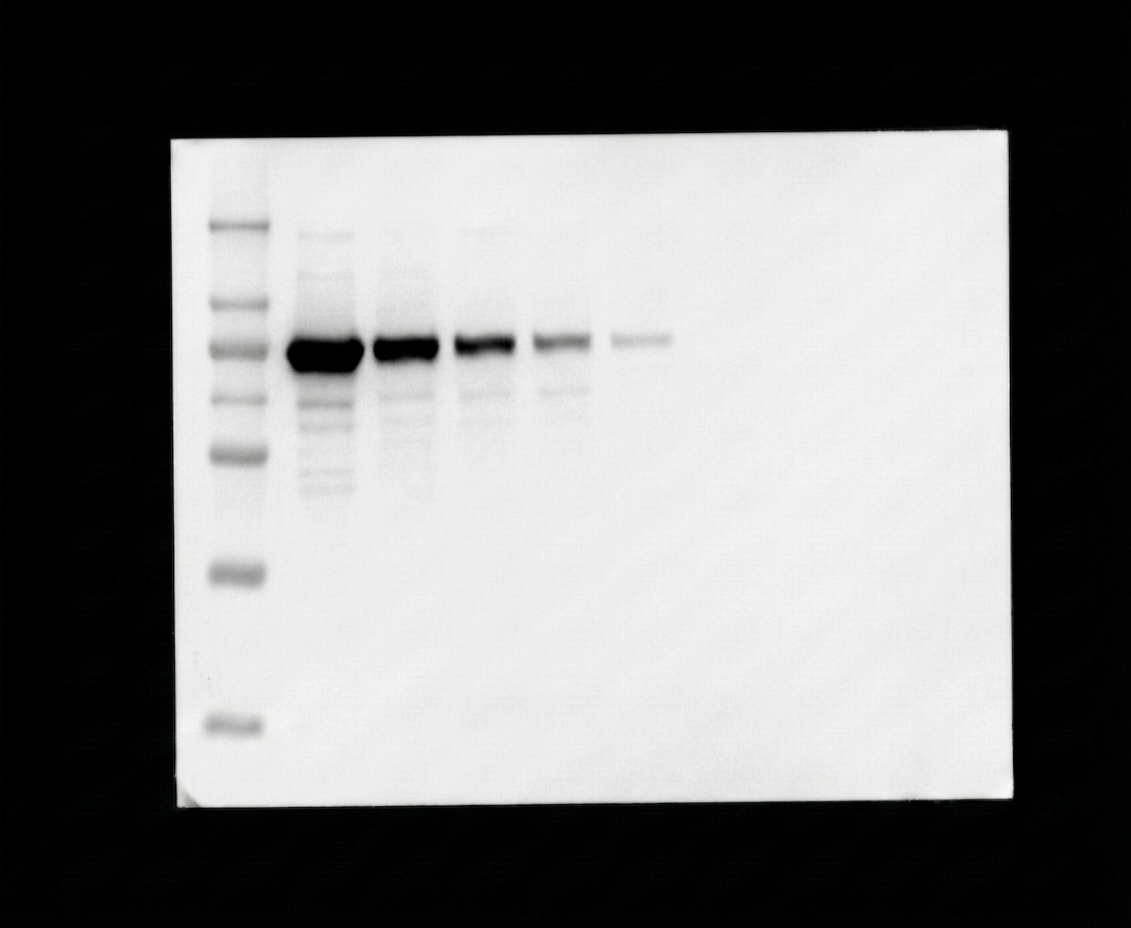

Supplement: S1 File — Three independent experiments (Rep 1, Rep 2, Rep 3) were performed using separately cultured cells. (ZIP) [file pone.0350841.s003.zip › data process/The second biological repetition/GRAS.tif]

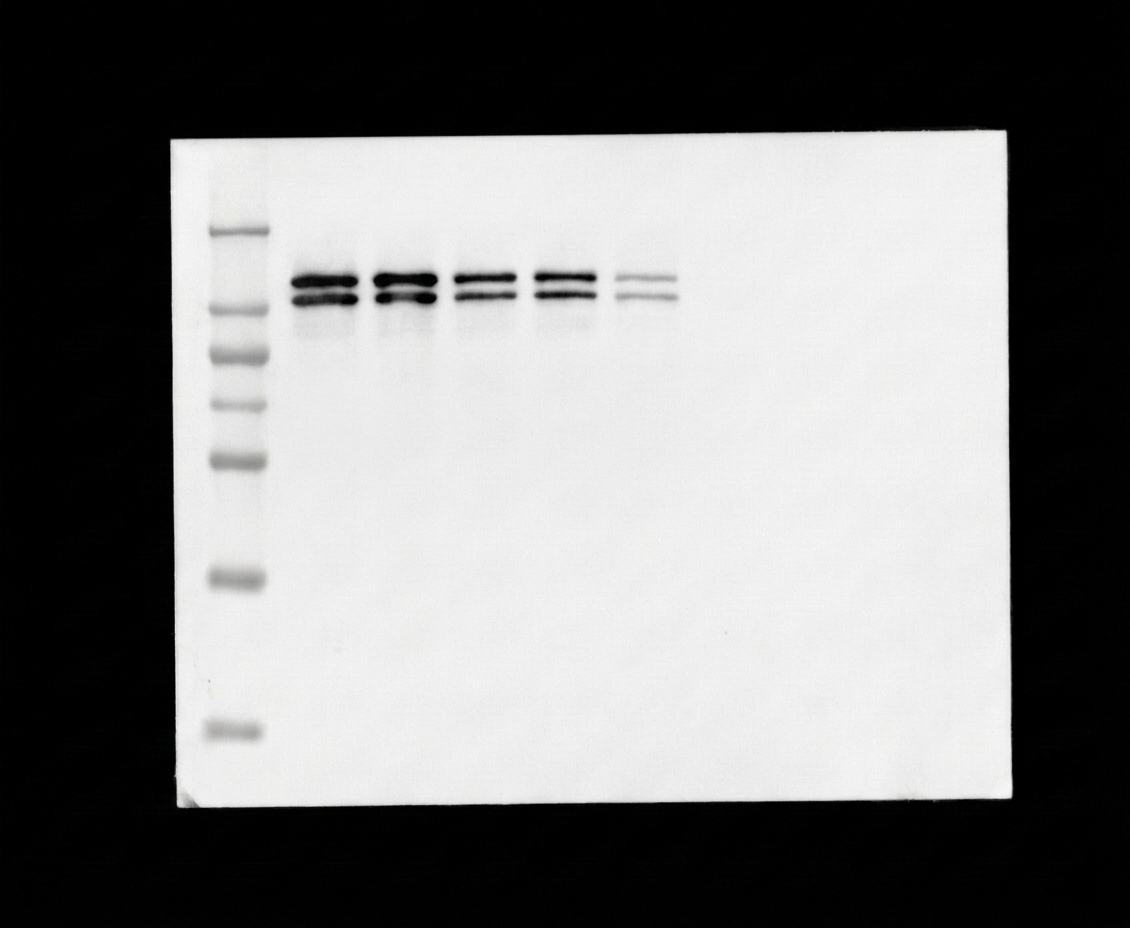

Supplement: S1 File — Three independent experiments (Rep 1, Rep 2, Rep 3) were performed using separately cultured cells. (ZIP) [file pone.0350841.s003.zip › data process/The second biological repetition/SHMT2.tif]

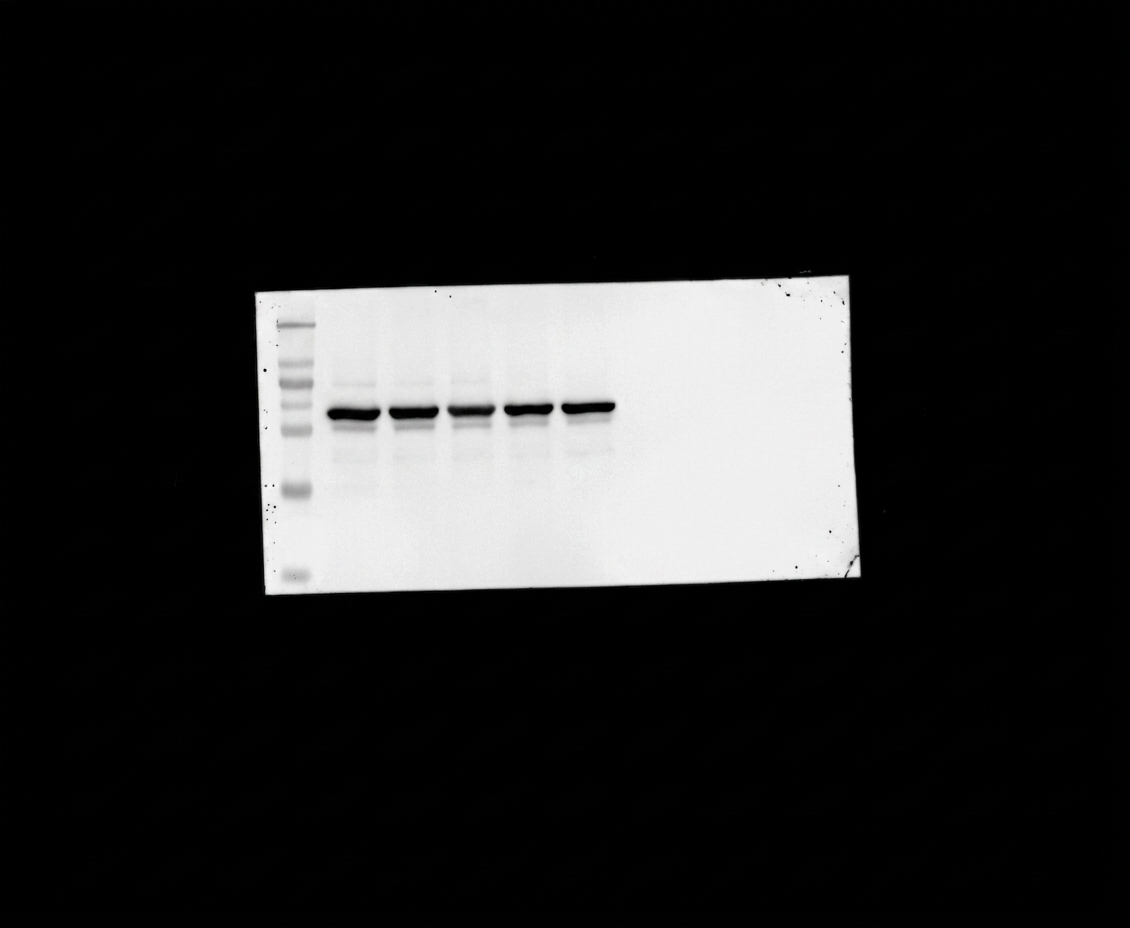

Supplement: S1 File — Three independent experiments (Rep 1, Rep 2, Rep 3) were performed using separately cultured cells. (ZIP) [file pone.0350841.s003.zip › data process/The third biological repetition/actin.tif]

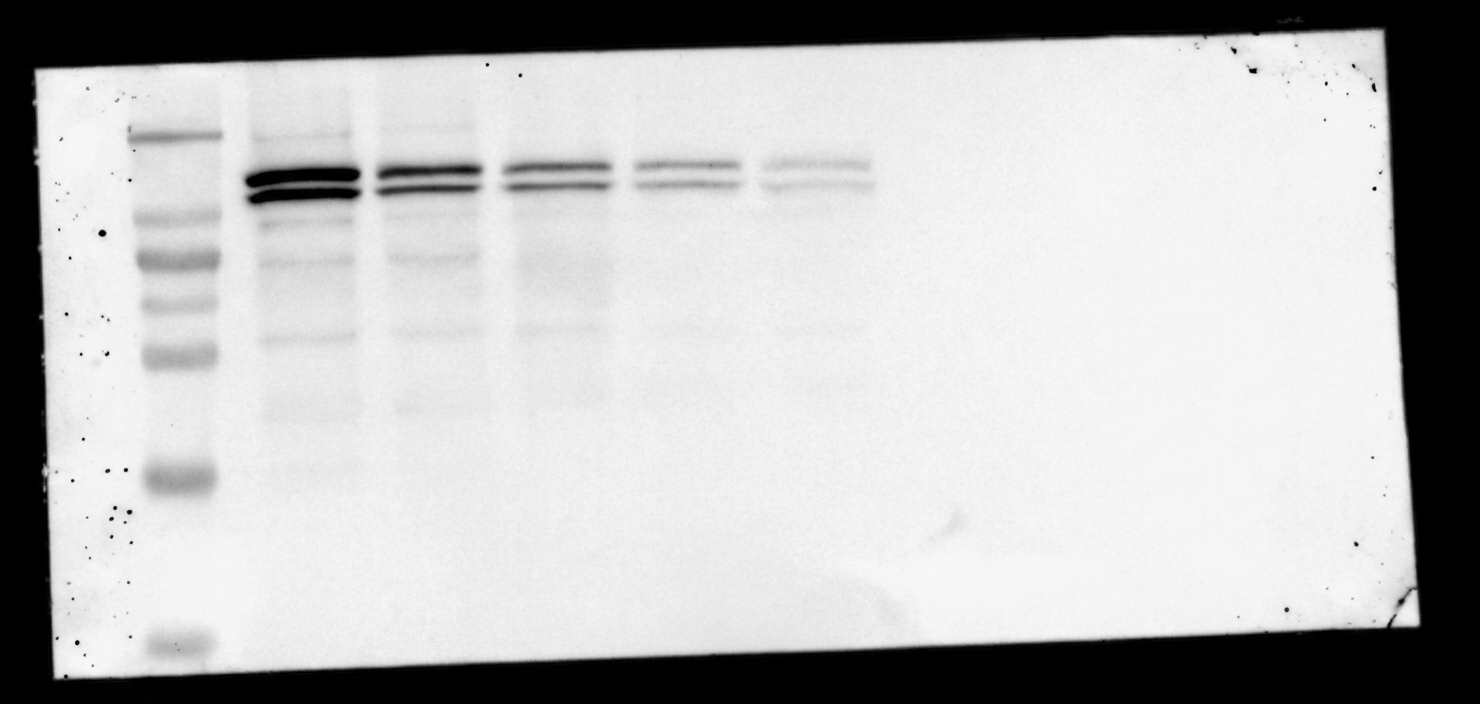

Supplement: S1 File — Three independent experiments (Rep 1, Rep 2, Rep 3) were performed using separately cultured cells. (ZIP) [file pone.0350841.s003.zip › data process/The third biological repetition/GRAS.tif]

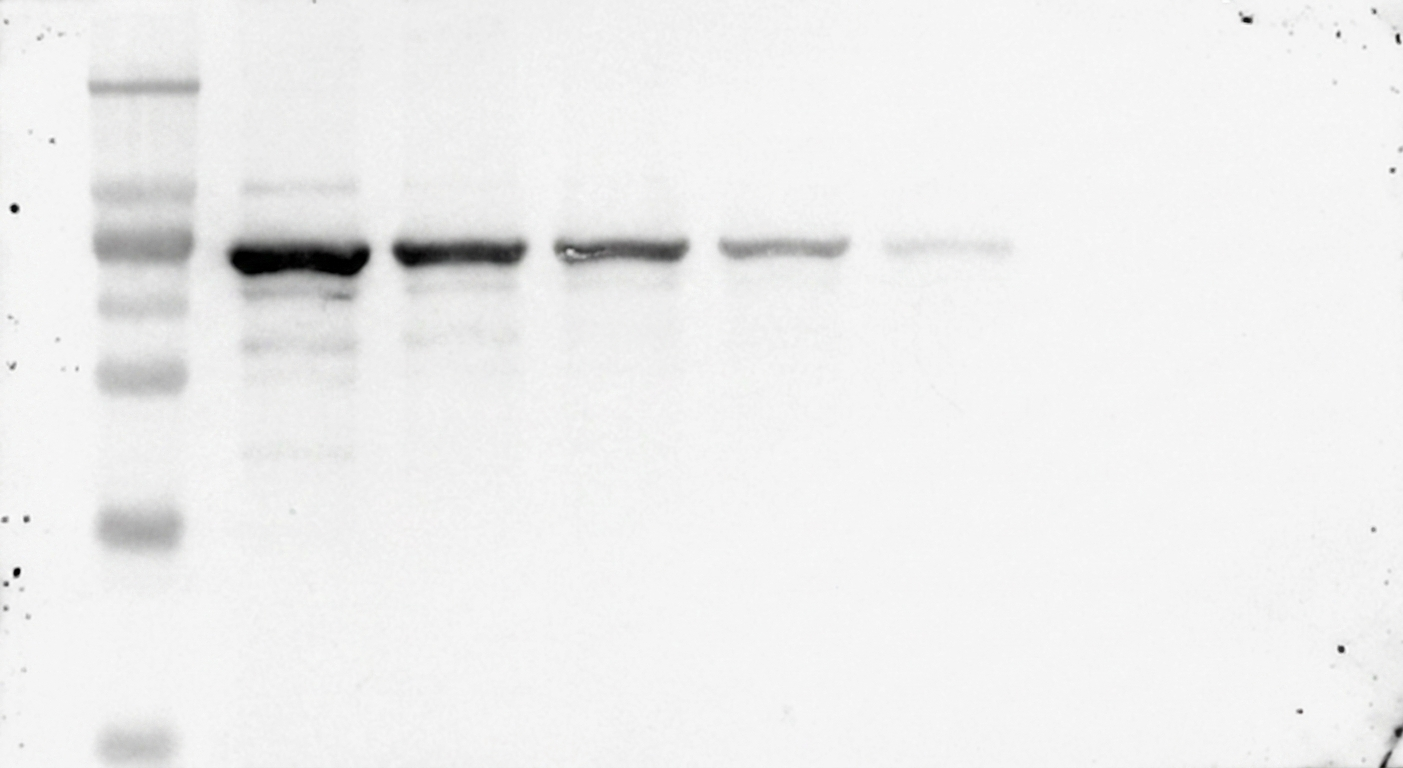

Supplement: S1 File — Three independent experiments (Rep 1, Rep 2, Rep 3) were performed using separately cultured cells. (ZIP) [file pone.0350841.s003.zip › data process/The third biological repetition/SHMT2.tif]

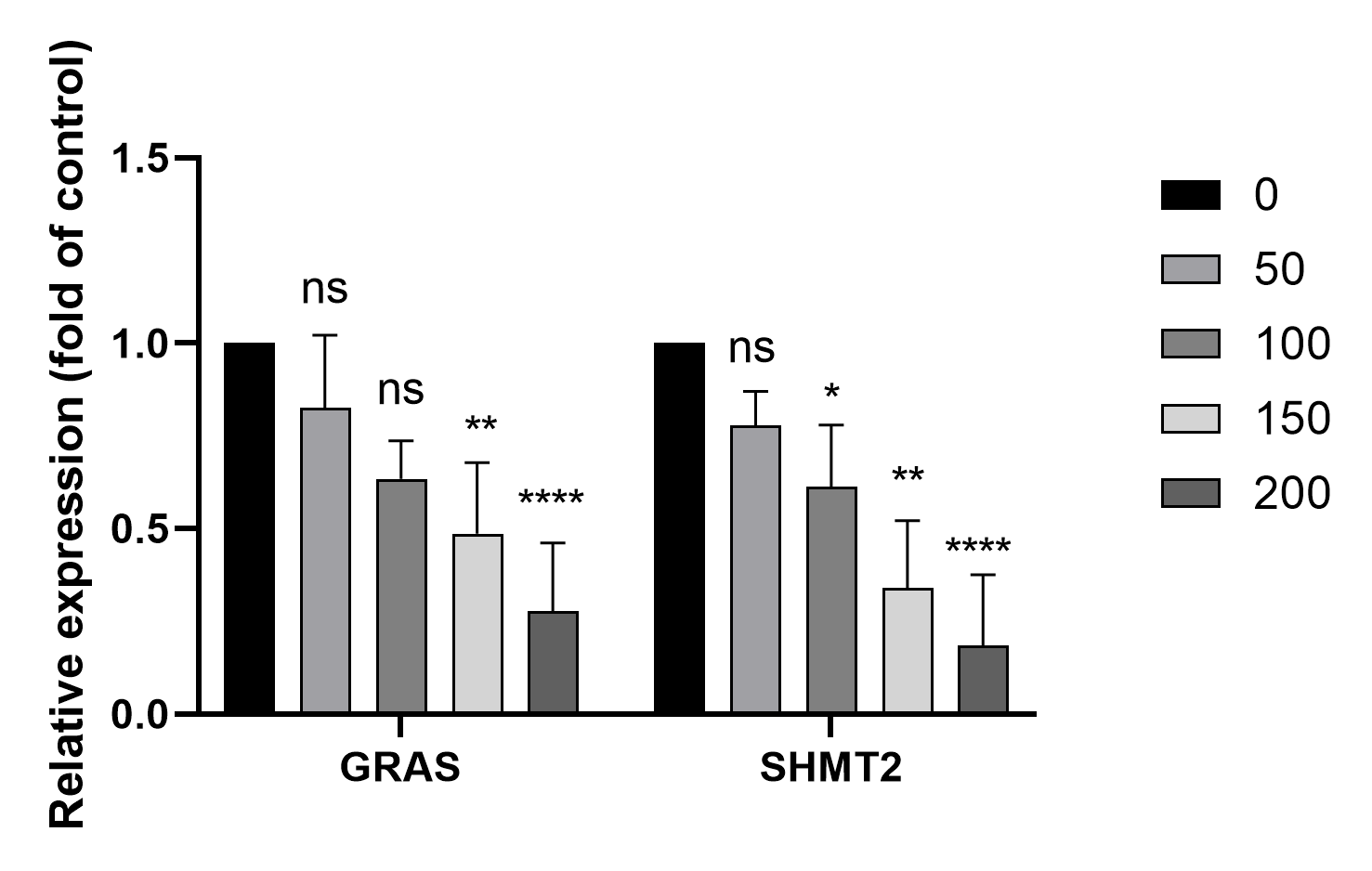

Supplement: S1 File — Three independent experiments (Rep 1, Rep 2, Rep 3) were performed using separately cultured cells. (ZIP) [file pone.0350841.s003.zip › data process/统计图.tif]

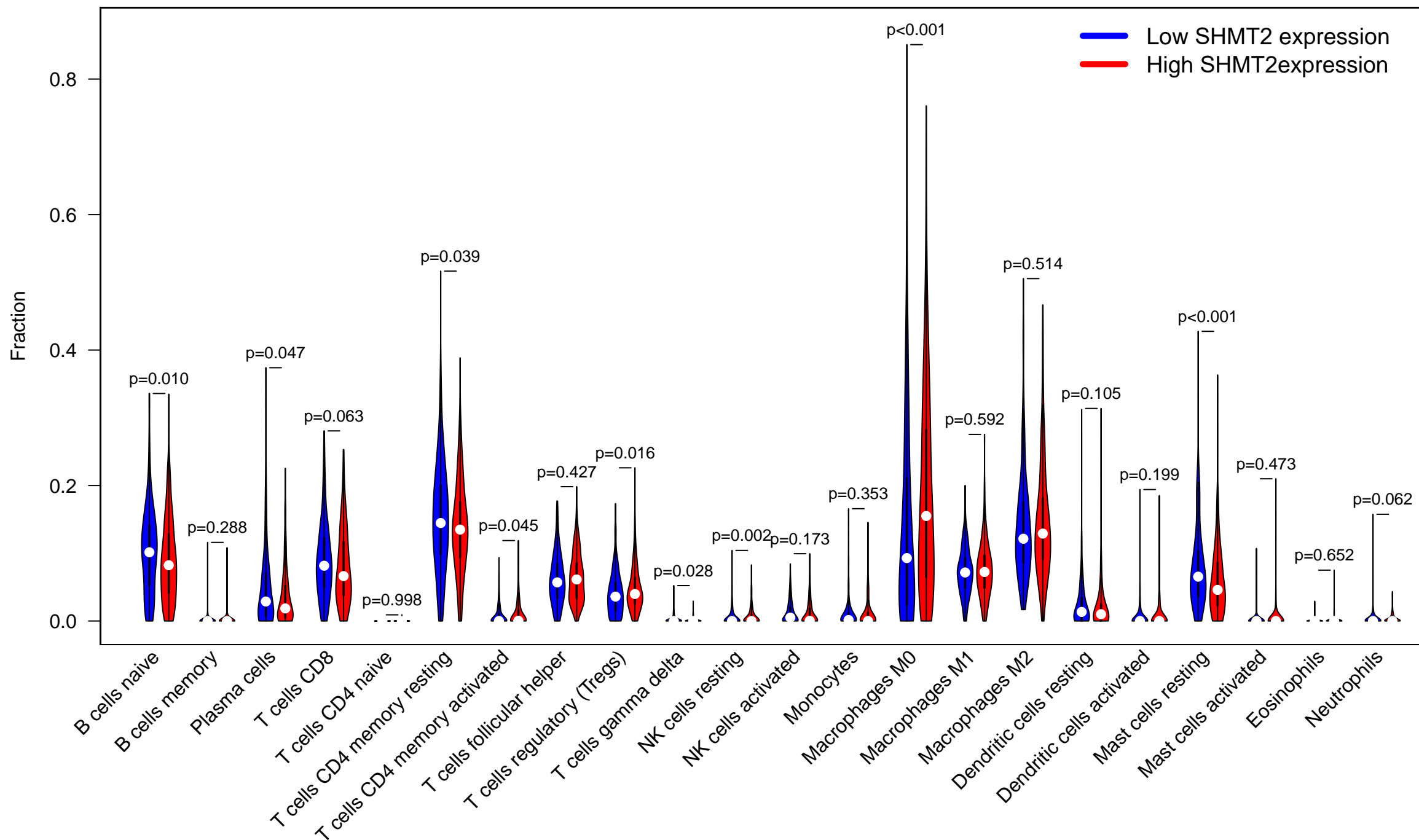

Supplement: S2 File — (ZIP) [file pone.0350841.s004.zip › Immune infiltration analysis and vilot plot/02.vioplotSHMT2/02.vioplotSHMT2/vioplot.pdf]

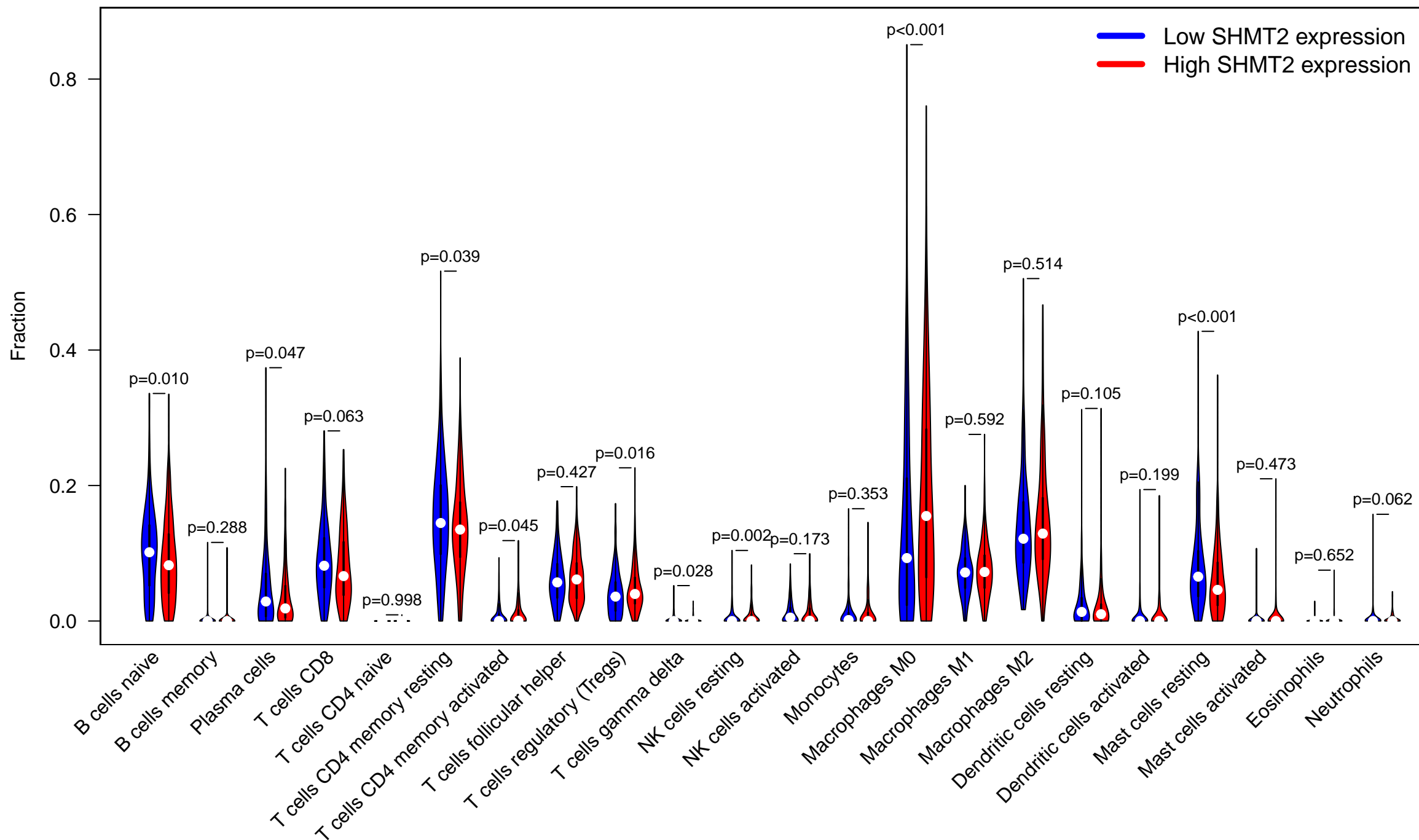

Supplement: S2 File — (ZIP) [file pone.0350841.s004.zip › Immune infiltration analysis and vilot plot/02.vioplotSHMT2/vioplot.pdf]

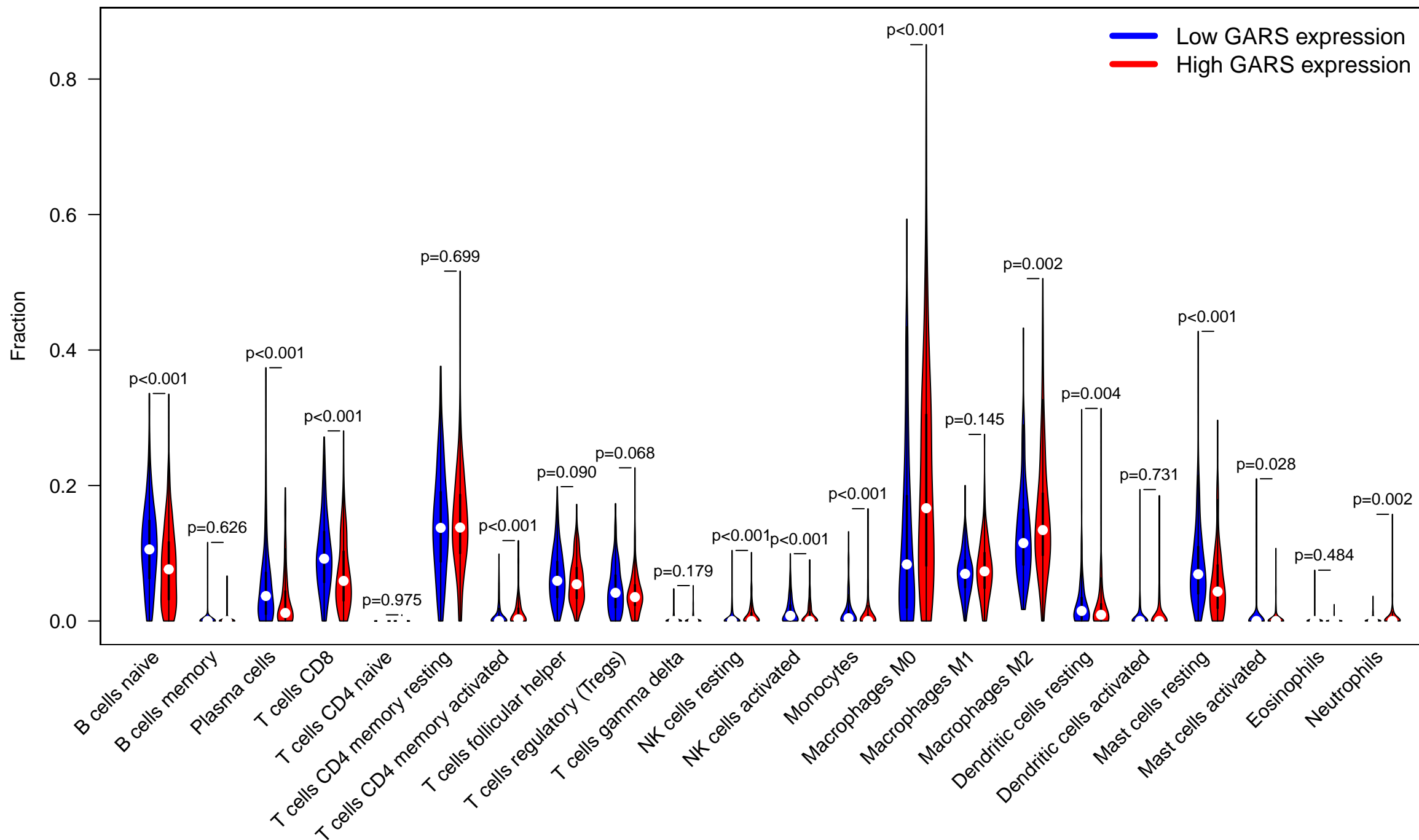

Supplement: S2 File — (ZIP) [file pone.0350841.s004.zip › Immune infiltration analysis and vilot plot/03.vioplotGARS/03.vioplotGARS/vioplot.pdf]
